# Correlation of Resting Elbow Angle with Spasticity in Chronic Stroke Survivors

**DOI:** 10.3389/fneur.2015.00183

**Published:** 2015-08-26

**Authors:** Minal Y. Bhadane, Fan Gao, Gerard E. Francisco, Ping Zhou, Sheng Li

**Affiliations:** ^1^Department of Physical Medicine and Rehabilitation, The University of Texas Health Science Center at Houston, Houston, TX, USA; ^2^NeuroRehabilitation Research Laboratory, The NeuroRecovery Research Center at TIRR Memorial Hermann Research Center, Houston, TX, USA; ^3^The University of Texas Southwestern Medical Center, Dallas, TX, USA; ^4^Guangdong Work Injury Rehabilitation Center, Guangzhou, China

**Keywords:** stroke, spasticity, resting angle, MAS, Tardieu

## Abstract

**Objective:**

To evaluate whether resting joint angle is indicative of severity of spasticity of the elbow flexors in chronic stroke survivors.

**Methods:**

Seventeen hemiparetic stroke subjects (male: *n* = 13; female: *n* = 4; age: 37–89 years; 11 right and 6 left hemiplegia; averaged 54.8 months after stroke, ranging 12–107 months) participated in the study. The number of subjects with modified Ashworth scale score (MAS) = 0, 1, 1+, 2, and 3 was 3, 3, 5, 3, and 3, respectively. In a single experimental session, resting elbow joint angle, MAS, and Tardieu scale score (Tardieu *R*1) were measured. A customized motorized stretching device was used to stretch elbow flexors at 5, 50, and 100°/s, respectively. Biomechanical responses (peak reflex torque and reflex stiffness) of elbow flexors were quantified. Correlation analyses between clinical and biomechanical assessments were performed.

**Results:**

Resting elbow joint angle showed a strong positive correlation with Tardieu *R*1 (*r* = 0.77, *p* < 0.01) and a very strong negative correlation with MAS (*r* = −0.89, *p* < 0.01). The resting angle also had strong correlations with biomechanical measures (*r* = −0.63 to −0.76, *p* < 0.01).

**Conclusion:**

Our study provides experimental evidence for anecdotal observation that the resting elbow joint angle correlates with severity of spasticity in chronic stroke. Resting angle observation for spasticity assessment can and will be an easy, yet a valid way of spasticity estimation in clinical settings, particularly for small muscles or muscles which are not easily measurable by common clinical methods.

## Introduction

Post-stroke spasticity is one of the most physically debilitating conditions that interfere with functional improvement (1, 2). Prevalence estimates of spasticity are highly variable, ranging from 20 to 46% (3–6). Spasticity significantly affects their quality of life, thus causing a significant burden for survivors and caregivers (2, 7).

Spasticity, commonly defined as “a motor disorder characterized by a velocity-dependent increase in tonic stretch reflexes (‘muscle tone’) with exaggerated tendon jerks, resulting from hyperexcitability of the stretch reflex, as one component of the upper motor neuron syndrome” (8). It can be easily recognized, but difficult to objectively quantify because of its multifactorial nature (1, 9). Measuring spasticity using reliable and valid tools is important for treatment planning rationale and also to evaluate treatment efficacy (10–13).

Clinical scales including the Ashworth (14, 15) and Modified Ashworth (MAS) (16) are commonly used for assessment of spasticity. But these scales do not capture other symptoms and signs of the upper motor neuron syndrome, such as co-contraction during movements or spasms (12, 17). The Tardieu Scale is considered as a more appropriate clinical measure of spasticity as it involves assessment of resistance to passive movement at both slow and fast speeds (18, 19). Even though reliability and validity of these clinical scales have been studied in many research studies, it is still controversial (16, 17, 20, 21). Laboratory tests, such as motorized stretching (22–25) and electrophysiological measurements, have higher accuracy but they are time consuming, expensive, and cannot be easily implemented in clinical environment (26–28). These approaches require specialized motor-driven mechanical systems that are not only space-prohibitive in a rehabilitation clinic, but require significant training and ongoing technical maintenance. Although these studies have given insight into neurophysiological aspects of spasticity, these techniques are unlikely to be widely adopted by clinics for routine use. Also except for commonly studied joints (elbow, wrist, knee, and ankle), it is difficult to apply these methods to spastic muscles in the neck and trunk areas, as well as those crossing the joint and changing their direction, e.g., posterior tibialis muscle. Hence, there is a clear need to explore alternative ways to incorporate assessment of spasticity into clinical practice.

It is a common clinical observation that altered resting posture of the trunk and joint correlates with spasticity of the respective muscles. Accordingly, the objective of this study was to evaluate whether resting joint angle is indicative of severity of spasticity of the elbow flexors in chronic stroke survivors by correlating resting joint angle with other frequently used clinical (MAS, Tardieu) and biomechanical methods for assessment of spasticity.

## Materials and Methods

### Subjects

Seventeen hemiparetic stroke subjects (age: 37–89 years; 11 right and 6 left hemiplegia; averaged 54.8 months after stroke, ranging 12–107 months) participated in the study. Table 1 displays characteristics of the subjects. Inclusion criteria were: (1) hemiplegia secondary to a single ischemic or hemorrhage stroke; (2) at least 6 months post-stroke; (3) elbow flexor spasticity of the impaired side less than 4 (rated by MAS); (4) able to understand and follow instructions related to the experiment; and (5) able to give informed written consent. The exclusion criteria were: (1) a history of multiple strokes or bilateral involvement; (2) presence of contracture that would limit full elbow range of motion on the impaired side. The number of subjects with MAS = 0, 1, 1+, 2, and 3 was 3, 3, 5, 3, and 3, respectively. The experiment was approved by the UTHealth Committee for the Protection of Human Subjects. All subjects gave written informed consent prior to participation.

**Table 1:** **Characteristics of stroke subjects (F: female, M: male, angle in degrees, fully extended position of the elbow was considered as 180°, age in years, post stroke months, ROM: range of motion)**.

ID	Arm	Age	Sex	Weight (lb)	Post stroke	Elbow MAS	Resting angle	Passive ROM	Active ROM	Tardieu angle *R*1
1	Right	57	F	187	65	1+	142	40–180	56–180	112
2	Right	67	M	272	30	**1+**	**150**	65–169	82–151	118
3	Left	68	M	176	50	**0**	**168**	52–180	Full	**180**
4	Left	59	F	130	16	1	166	55–180	60–180	160
5	Right	75	M	195	93	1	170	48–180	Full	135
6	Right	50	M	180	26	0	175	70–180	Full	180
7	Left	89	M	226	71	**1+**	**150**	60–180	90–170	130
8	Right	62	M	200	93	**0**	**168**	50–180	50–170	**180**
9	Left	70	M	186	74	1+	132	60–152	None	122
10	Right	37	M	205	29	3	110	60–142	None	98
11	Right	65	M	135	97	**3**	**120**	84–154	None	**100**
12	Left	54	M	185	49	**3**	**120**	30–170	None	**100**
13	Right	54	M	230	41	2	136	42–170	None	120
14	Right	52	M	182	13	2	138	52–280	None	122
15	Right	76	M	214	12	1	160	55–170	55–160	110
16	Left	49	F	115	67	2	150	54–180	70–141	95
17	Right	50	F	144	107	1+	125	56–180	Full	110

*Bold numbers reflect patients with same MAS, resting, and/or Tardieu *R*1 angle. This makes less data points in Figure 4 than the actual number of cases*.

### Procedure

The study had two sets of measurements, including clinical assessment and biomechanical measurements. The following clinical assessments were performed on each subject: (1) passive range of motion; (2) active range of motion; (3) MAS: resistance to passive elbow flexion was assessed by stretching the muscle at a moderate speed and scoring the resistance using MAS; (4) Tardieu scale (Tardieu *R*1): angle measured at a fast speed when the muscle reaction was first felt (if there was no muscle reaction, Tardieu angle was considered as 180°); and (5) resting angle (*R*): to evaluate gravity effect. The fully extended position of the elbow was defined as 180°. For clinical measurements, subjects were explicitly instructed to stand and relax the affected arm as much as they could. The measurements were taken after they were standing upright still for at least 1 min. Subjects were allowed to take support of a person/chair/walker/cane with the unaffected arm.

**Figure 1:**
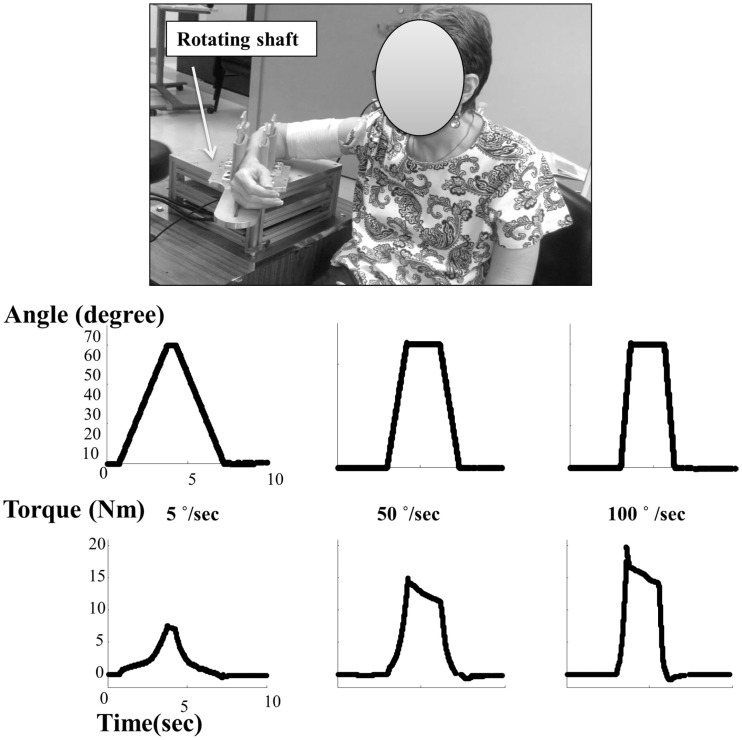
**Experimental setting and representative torque–angle profiles at different speeds**.

Biomechanically, responses to constant velocity stretch of elbow flexors were measured. We adopted our previous experimental setup (29). The subjects were seated on a height adjustable chair. The arm to be tested was secured firmly on a customized apparatus with a servomotor. The shoulder was positioned at ∼45° of abduction and 30° of flexion. The center of the elbow joint was aligned with the axis of rotation of the servo motor. The forearm was firmly secured using four vertical plates at the proximal and distal forearm (Figure 1). The subjects were instructed to naturally relax the wrist, hand, and fingers without additional support during external stretching. This arrangement prevented translation and rotation of the arm. The other arm of the subject rested alongside the body.

### Experimental protocol

Subjects completed a single session during which the elbow joint of the impaired side was passively stretched at different velocities. Only the affected side was tested. A total range of 60° stretch was utilized with the end position (*E*) at 10° beyond the resting angle (*R*) (*E* = *R* + 10). The initial position (*I*) was *I* = *E* − 60°. For patients with resting angle close to or more than 170°, the end position (*E*) was considered to be at 180° making the initial position (*I*) of 120°. Goniometer was used to position arm at the initial angle. The end joint angle was set at 180°, if the resting angle was 170° or greater. The trial began with the elbow at the initial position (*I*), and then a constant velocity extension movement was imposed at the elbow until the elbow reached the predetermined end position (*E*). The elbow was then held in the end position for 2 s and returned to the initial position at the same velocity. A rest period of about 30 s was allowed between trials to allow adequate recovery and to minimize the influence of stretch history on the response to the subsequent stretch. Subjects were instructed to relax during the trials, neither supporting nor opposing the joint extension. Three velocities of 5, 50, and 100°/s were used with three trials at each velocity.

Torque was measured with a torque sensor (Model TRS 500, Transducers Techniques, CA, USA). An angular motion recorded using encoder (HD FHA-25C-50-US250, Standard Incremental, 2500 pulses per revolution). All signals were digitized at 1000 samples/s on a PC computer with a data acquisition board (National Instruments, Austin, TX, USA) using custom LabView software (National Instruments). Data was saved for offline analysis using a customized MATLAB (The MathWorks Inc.) program.

### Data analysis

Angle and torque signals were analyzed to determine biomechanical response of stretch in the spastic elbow flexors. The torque signal was filtered using a 100-point moving window median filter to remove outlier noise evident in the raw data. Mean of initial 100 ms data was subtracted from complete data set to remove any DC bias. To avoid data variation as a result of anthropometry spread between subjects, torque was normalized by individual body weight. To characterize the pattern of the response, average torque was calculated across all three trials for each speed (Figure 1). For each subject, peak torque was calculated for all speeds between the start and end of rotation. Reflex torque was calculated by subtracting torque response at 5°/s from those at 50 and 100°/s (23). The reflex stiffness was computed by finding slope from the linear regression of reflex torque–angle profile. The limits to finding slope were decided to be 25 and 75% of the maximum torque for a given trial (23).

#### Statistics

Linear regression analysis was performed on torque and stiffness data with resting angle, Tardieu *R*1, and MAS. Correlations between clinical assessment (MAS, Tardieu *R*1, and resting angle) and biomechanical measures (peak reflex torque and reflex stiffness) were analyzed using Spearman’s coefficient (*r*). Furthermore, a repeated measures one-way analysis of variance (ANOVA) was used to analyze the effect of velocity on peak torque with a factor of VELOCITY. Statistical significance was set at *p* < 0.05.

## Results

### Velocity-dependent responses

**Figure 2:**
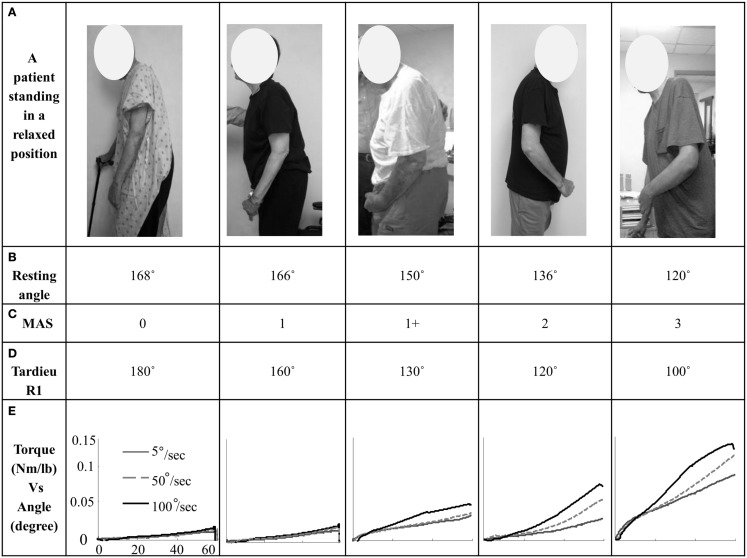
**(A)** A stroke patient (representing each MAS score group) standing in a relaxed position; **(B)** resting angle in degrees; **(C)** MAS score; **(D)** Tardieu *R*1 angle in degrees; **(E)** torque–angle response (mean of three trials) for the speeds 5, 50, and 100°/s for each subject of a representing MAS score group.

**Figure 3:**
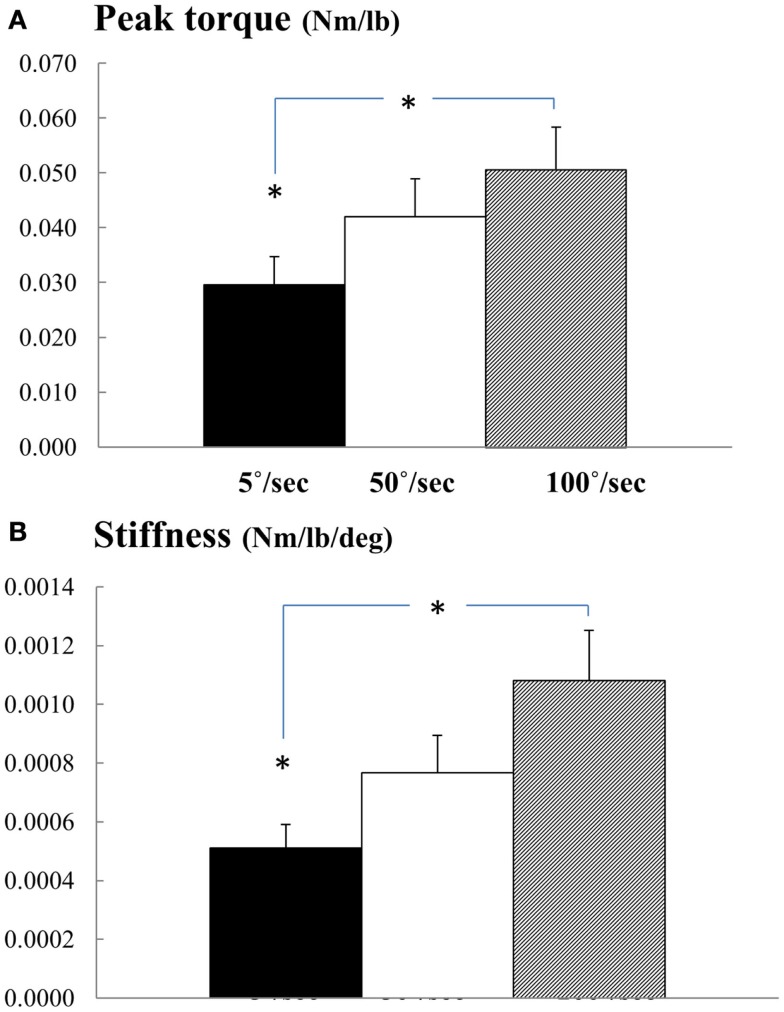
**(A)** Normalized peak torque and **(B)** stiffness at three speeds. Mean and SEs are plotted. * represents statistical significance.

Overall, we observed velocity-dependent mechanical responses. Figure 1 shows representative torque–angle profile recorded for all three speeds. Figure 2 demonstrates comparative torque–angle data for patients representing each of the five MAS levels. Photos of patients in standing positions used for resting joint angle measurement are shown in first row. The torque–angle curve increased sharply at the beginning of the stretch due to inertial effects and then settled into a constant slope. Effect of speed on torque amplitude was apparent in Figures 1 and 2. A repeated one-way ANOVA showed a main effect of VELOCITY for peak torque response (*F*[3,14] = 15.63, *p* < 0.0001) (Figure 3A). Figure 3B shows the direct relationship between stiffness in elbow flexors and velocity. Similarly, there was a main effect of VELOCITY (*F*[3,14] = 12.68, *p* = 0.0002).

### Correlations between clinical and biomechanical assessments

**Figure 4:**
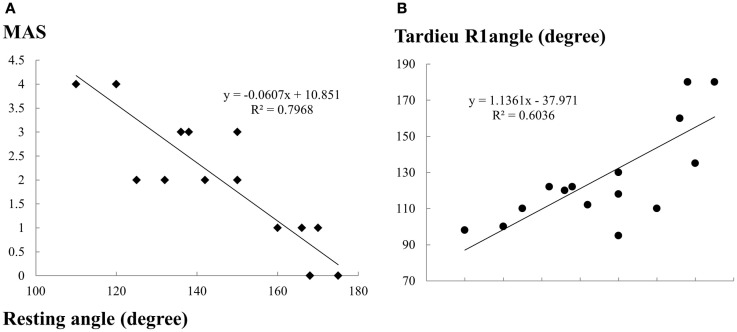
**Correlations between the resting angle and clinical measurements (A) MAS and (B) Tardieu *R*1 angle**. Because of same measures for some cases, there are less data points (cases) on the figure than the actual number of cases. Refer to Table 1 for details.

The resting angle showed a strong positive correlation with the Tardieu *R*1 angle (*r* = 0.77, *p* < 0.01) and a strong negative correlation with MAS (*r* = −0.89, *p* < 0.01) (Figure 4). The resting angle also showed strong correlations with peak reflex torque (*r* ranged from −0.639 to −0.700, *p* < 0.01) and reflex stiffness (*r* ranged from −0.716 to −0.763, *p* < 0.01) (Table 2). All clinical measures, the resting angle (*r* ranged from −0.63 to −0.76, *p* < 0.01), MAS (*r* ranged from 0.79 to 0.84, *p* < 0.01), and the Tardieu *R*1 angle (*r* ranged from −0.58 to −0.63, *p* < 0.05) showed strong correlations with biomechanical measurement (peak reflex torque and reflex stiffness) (Table 2).

**Table 2:** **Correlations among resting angle, Tardieu *R*1 angle, MAS and biomechanical parameters: peak reflex torque and reflex stiffness**.

Parameters	Resting angle	Tardieu	MAS
Resting angle			
Tardieu *R*1 angle	0.777**		
MAS	−0.893**	−0.855**	
Peak reflex torque 50°/s	−0.639**	−0.631**	0.695**
Peak reflex torque 100°/s	−0.700**	−0.601*	0.764**
Reflex stiffness 50°/s	−0.716**	−0.589*	0.763**
Reflex stiffness 100°/s	−0.763**	−0.606**	0.841**

*The numbers in the table refer to the regression coefficients (*r*)*.

*p < 0.05.

**p < 0.01.

## Discussion

Given the constraints of a clinical environment, technique for spasticity assessment must be clinically valid and easy to implement. In this study we evaluated relation between the severity of spasticity at the elbow joint and the resting joint angle. The results of the biomechanical tests in this study provide experimental evidence that resting joint angle can be used to estimate post-stroke spasticity.

### Strong correlations between biomechanical and clinical parameters

Our results of velocity-dependent peak torque and reflex stiffness were consistent with previous reports (15, 22–24, 27). There were controversial reports of relations between biomechanical measures and clinical scale (15, 26–28, 30, 31). In a study of 14 stroke subjects, reflex stiffness measured at 90° of elbow flexion for all subjects had very weak correlation (*r* = 0.2) with MAS (15). In contrast, when passive stretches were applied to the full comfortable range of motion of the elbow joint, reflex torque and stiffness had strong correlations with the Ashworth scale score in a group of 16 stroke subjects (27). Our findings were consistent with the latter study (27, 30), showing strong correlations between biomechanical measures and clinical measures. Though commonly used as in the above cited studies, we are aware of the limitation of the use of parametric statistics for non-linear data, such as the MAS and Tardieu scales.

Biomechanical measures of spastic muscles are length-dependent in stroke subjects (15, 24). Measurement at a standardized joint angle does not reflect pathological state of patients with different severities of spasticity. The resting joint angle may represent a reference for the new equilibrium point of the neuromuscular system after stroke (25). Thus, biomechanical measures obtained from passive stretch with reference to the resting angle are comparable across subjects and show strong correlations with other clinical measures.

### Insight into pathophysiology of spasticity

Despite the advances in the treatment of spasticity, there are several gaps in research and clinical practice, foremost of which is the relative deficiency of knowledge of the pathophysiology of spasticity. It is well accepted that there is hyperexcitability of the stretch reflex in spasticity (32–36). Excitability of the spinal stretch reflex arc is maintained by a balanced descending regulation and normal intraspinal processing. Therefore, stretch reflex hyperexcitability in post-stroke spasticity could be mediated by two categories of mechanisms: abnormal descending regulations and/or abnormal intraspinal processing of stretch reflex. Accumulated evidence suggest that abnormal intraspinal processing likely results from plastic rearrangement secondary to abnormal descending regulation; in contrast, imbalanced descending inhibitory and excitatory inputs, particularly reticulospinal hyperexcitability, as a result of unmasking after stroke is the primary underlying mechanism for spasticity [see reviews in Ref. (37)]. Acoustic startle reflex is a brainstem reflex which is primarily medicated by the reticulospinal pathway. In a recent study, reticulospinal hyperexcitability, as reflected by exaggerated acoustic startle reflex responses, was only seen in stroke survivors with elbow flexor spasticity, but not in those without (flaccid or fully recovered) (38). Furthermore, the reticulospinal pathway also plays an important role in maintaining joint position and posture against gravity (39). Altered reticulospinal excitability and its anti-gravity effect could lead to a new neuromuscular balance, reflecting a shift in reference configuration after stroke (25, 40). This new balance could be reflected by a change in the resting angle of a joint. The results of high correlations between severity of spasticity and resting joint angle, thus, suggest that spasticity is strongly related to reticulospinal hyperexcitability and its anti-gravity effects.

### Clinical significance of resting joint angle

Our results revealed that there existed overall strong correlations between resting joint angle and other frequently used clinical (MAS, Tardieu *R*1 angle) and biomechanical (stretch reflex response) measurements. As such, our study provides evidence that resting joint angle correlates with severity of post-stroke spasticity in elbow flexors. This finding is clinically useful in that the resting angle could be easily and objectively quantified. No subjective interpretation is involved, for example, a subjective feeling of “catch” in other clinical scales. Therefore, resting joint angle could be used as objective outcome measures for treatment, e.g., before and after botulinum toxin injections. Resting angle is particularly helpful for muscles which are not easily measurable by common clinical methods, such as spastic sternocleidomastoid muscles. However, the results may not be applicable to weight-bearing joints, where ground reaction force may alter joint position independent of spasticity’s effect. Soft tissue contractures are often present in spastic muscles (41). They may partially account for the joint abnormality as well. Another limitation of this study is a small sample size. Future study with a large sample size is needed.

To summarize, our study provides experimental evidence for anecdotal observation that resting elbow joint angle correlates with severity of spasticity in chronic stroke.

## Conflict of Interest Statement

The authors declare that the research was conducted in the absence of any commercial or financial relationships that could be construed as a potential conflict of interest.
